# Age-Dependent Hepatic UDP-Glucuronosyltransferase Gene Expression and Activity in Children

**DOI:** 10.3389/fphar.2016.00437

**Published:** 2016-11-16

**Authors:** Elizabeth Neumann, Huma Mehboob, Jacqueline Ramírez, Snezana Mirkov, Min Zhang, Wanqing Liu

**Affiliations:** ^1^Department of Medicinal Chemistry and Molecular Pharmacology, College of Pharmacy, Purdue UniversityWest Lafayette, IN, USA; ^2^Department of Biochemistry, University of AgricultureFaisalabad, Pakistan; ^3^Section of Hematology and Oncology, Department of Medicine, The University of ChicagoChicago, IL, USA; ^4^Department of Statistics, College of Science, Purdue UniversityWest Lafayette, IN, USA; ^5^Beijing Institute for Brain Disorders, Capital Medical UniversityBeijing, China

**Keywords:** UDP-glucuronosyltransferase, liver, children, age, ontogeny

## Abstract

UDP-glucuronosyltransferases (UGTs) are important phase II drug metabolism enzymes. The aim of this study was to explore the relationship between age and changes in mRNA expression and activity of major human hepatic UGTs, as well as to understand the potential regulatory mechanism underlying this relationship. Using previously generated data, we investigated age-dependent mRNA expression levels of 11 hepatic UGTs (*UGT1A1, UGT1A3, UGT1A4, UGT1A5, UGT1A6, UGT1A9, UGT2B4, UGT2B7, UGT2B10, UGT2B15*, and *UGT2B17*) and 16 transcription factors (*AHR, AR, CAR, ESR2, FXR, GCCR, HNF1a, HNF3a, HNF3b, HNF4a, PPARA, PPARG, PPARGC, PXR, SP1*, and *STAT3*) in liver tissue of donors (*n* = 38) ranging from 0 to 25 years of age. We also examined the correlation between age and microsomal activities using 14 known UGT drug substrates in the liver samples (*n* = 19) of children donors. We found a statistically significant increase (nominal *p* < 0.05) in the expression of *UGT1A1, UGT1A3, UGT1A4, UGT1A5, UGT1A6*, *UGT2B7*, and *UGT2B17*, as well as glucuronidation activities of serotonin, testosterone, and vorinostat during the first 25 years of life. Expression of estrogen receptor 1 and pregnane X receptor, two strong UGT transcriptional regulators, were significantly correlated with both age and *UGT* mRNA expression (*p* ≤ 0.05). These results suggest that both *UGT* expression and activity increase during childhood and adolescence, possibly driven in part by hormonal signaling. Our findings may help explain inter-patient variability in response to medications among children.

## Introduction

UDP-glucuronosyltransferases (UGTs) are a family of enzymes that make significant contributions to the hepatic metabolism of exogenous (e.g., drugs) and endogenous (e.g., bilirubin, testosterone, serotonin) substances, as well as numerous environmental pollutants, dietary chemicals, and chemical carcinogens. They also metabolize phase I oxidation products. UGTs catalyze the transfer of glucuronic acid from UDP glucuronic acid, causing the compounds or metabolites to undergo renal and biliary excretion. Drugs administered for therapeutic purpose exhibit variability in metabolism due to differing function and expression of these genes (Radominska-Pandya et al., 1999).

The regulation of UGT enzyme expression is complex and involves *cis*-factors (i.e., polymorphisms), *trans*-factors [such as transcription factors (TFs) and nuclear receptors (NRs)], miRNA targeting, and other epigenetic regulating factors (Bigo et al., 2013; Dluzen et al., 2014; Liu et al., 2014; Oda et al., 2014). In the liver, transcription levels of UGTs help determine their activity (Liu et al., 2014). Thus far, numerous polymorphisms have been identified that regulate UGT gene transcription. In addition, the tissue-specific and ligand activated TFs and NRs play a primary role in constitutive and inducible expression of UGTs by binding to *cis*-regulatory elements (CREs) (Liu et al., 2014). These regulators include constitutive androstane receptor (CAR), pregnane X receptor (PXR), hepatocyte nuclear factor 4 alpha (HNF4a), xenobiotic-activated aryl hydrocarbon receptor (AhR), and estrogen receptor (ESR1) (Bigo et al., 2013; Kalthoff et al., 2013; Hu et al., 2014; Liu et al., 2014). The inter-individual variability in expression of UGT family members contributes to the variability in response and toxicity of many medications.

Among children, however, the ontogeny of drug-metabolizing enzymes is paralleled by maturation of organ systems, and has profound effects on drug disposition in addition to being a principal factor for age-associated changes in drug clearance especially in fetal life and in the first months of postnatal life (Johnson, 2003; Blake et al., 2005; Dotta and Chukhlantseva, 2012; Fanni et al., 2014). Therefore, both age and genetic variation confer the variability in activity and expression of drug metabolizing enzymes among children. Ontogeny of several UGT enzymes has been explored with limited amounts of liver samples. It has been long found that several of the UGT enzymes experience changes in expression and/or activity from early gestational age and may not reach adult levels (Onishi et al., 1979; Leakey et al., 1987; Zaya et al., 2006; Miyagi and Collier, 2007, 2011; Miyagi et al., 2012; Divakaran et al., 2014). However, most previous studies were limited by fragmented data from different sample sets, which made it difficult to compare data between studies. The constant change of *UGT* gene expression and activity in human livers across childhood and adolescence has not been comprehensively investigated. In addition, while ontogenic *UGT* expression has been observed, no study has aimed at understanding the molecular mechanism underlying the age-related gene regulation.

We hypothesize that an empirical, pathway-based analysis of the age-dependent expression and activity of hepatic UGTs will allow us to perform a detailed comparison between these genes. We also postulate that hormonal regulation of *UGTs* may be a major reason for their age-dependent expression. This study aims to comprehensively examine the pattern of age-dependent mRNA expression and microsomal activities of all major hepatic *UGT* isoforms in human livers from individuals between 0 and 25 years old. Meanwhile, in order to understand the regulatory mechanism of UGT ontogeny, we also aim to examine the interrelationship between age and mRNA expression of 16 *UGT* regulatory TFs, and between the expression of relevant TFs (*ESR1* and *PXR*) and all *UGTs*. Such information is significant since it may influence therapeutic choices and dosing recommendations in the pediatric population.

## Materials and Methods

### Liver Tissue Samples and Data Collection

Human liver tissue samples (*n* = 38, 0–25 years old, 10 females, 28 males) from donor organs unsuitable for transplantation were described in previous studies (Gamazon et al., 2013; Liu et al., 2014). All donors are of Caucasian origin. The mRNA expression level of genes including 11 hepatic *UGTs* (*UGT1A1, UGT1A3, UGT1A4, UGT1A5, UGT1A6, UGT1A9, UGT2B4, UGT2B7, UGT2B10, UGT2B15*, and *UGT2B17*) and 16 TFs (*AHR, AR, CAR, ESR2, FXR, GCCR, HNF1a, HNF3a, HNF3b, HNF4a, PPARA, PPARG, PPARGC, PXR, SP1*, and *STAT3*) had been previously quantified with real-time PCR (Liu et al., 2014). Among these livers (*n* = 38), 19 were previously characterized for liver microsomal glucuronidation activity using 14 UGT substrates [substrates and major UGTs involved in their metabolism: SN-38 (UGT1A1, UGT1A9, UGT1A3, and UGT1A6), bilirubin (UGT1A1), thyroxine (UGT1A3 and UGT1A1), serotonin (UGT1A6), flavopiridol (UGT1A9), mycophenolic acid (UGT1A9), *S*-oxazepam (UGT2B15, UGT2B7, UGT1A1, and UGT1A6), testosterone (UGT2B17 and UGT2B15), epirubicin (UGT2B7), morphine (UGT2B7, UGT1A1, UGT1A3, UGT1A6, UGT1A8, UGT1A9, and UGT1A10), anastrozole (UGT1A4, UGT1A3, and UGT2B7), imipramine (UGT1A4 and UGT2B10), acetaminophen (UGT1A9, UGT1A1, UGT2B15, UGT1A6 and UGT2B7), and vorinostat (UGT2B17, UGT2B7, and UGT1A9)] (Liu et al., 2014). Data were obtained from a previously published study (Liu et al., 2014) and used for subsequent analyses. This study was originally approved by the Institutional Review Boards (IRBs) of The University of Chicago and Purdue University.

### Statistical Analysis

Age-related gene expression can be non-linear, to determine the best model to assess age-dependent *UGT* gene expression or activity, we first investigated the polynomial regression including the quadratic term of age (age^2^) for all *UGT* genes. Interestingly, age^2^ showed a marginally significant effect only on *UGT1A1* gene (*p* = 0.01), and the significance disappeared after controlling multiple comparison. For all other *UGT* genes, the raw p values of the effects of age^2^ range from 0.08 to 0.97. Given all these results, the linear model was employed for the final analysis as presented in the manuscript.

Correlation between age and expression of each of the *UGT* genes and glucuronidation activities were then tested using a linear regression model by including sex as a covariate. Correlation between mRNA expression levels of *UGTs* and TFs as well as between expression of *UGTs* and their substrates’ glucuronidation rates were performed based on Pearson’s correlation. With the transformed data, the normality assumption is satisfied. Although the sample size is relatively small, the relationship is approximately linear as tested above. Therefore, we prefer the more powerful Pearson’s correlation over the non-parametric test in our analysis.

The Benjamini–Hochberg’s false discovery rates (FDR) were used to control multiple testing. An FDR ≤ 0.05 was used as cut-off to correct for multiple testing. A nominal *p* ≤ 0.05 indicates a “suggestive” correlation. Statistical analyses were carried out using SPSS 23.0 (SPSS, Inc., Chicago, IL, USA), and data were plotted using Graphpad Prism 7.0 (GraphPad Prism, La Jolla, CA, USA).

## Results

### Correlation between Age and UGT mRNA Expression

**FIGURE 1:**
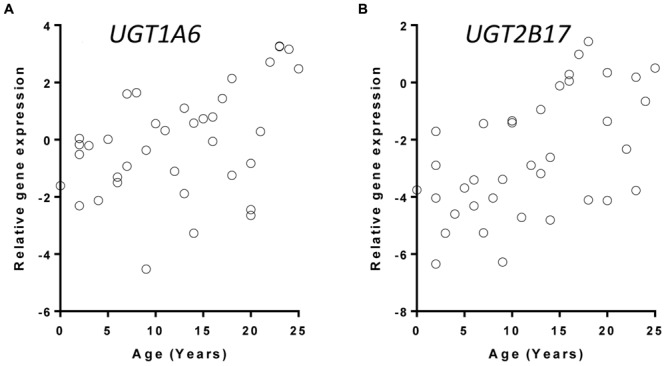
**Correlation between age and *UGT* gene expression with *UGT1A6* (A) and *UGT2B17* (B) as examples.** *UGT* gene expression were quantified with real-time PCR and normalized to the expression of beta-actin gene. Data were plotted as the +log2 transformed relative ratios (UGT/housekeeping gene).

The relationship between *UGT* gene expression and age was assessed using a linear regression model controlling for gender information. Of the 11 hepatic UGTs quantified using real-time PCR, 6 (*UGT1A1, UGT1A3, UGT1A4, UGT1A5, UGT1A6, UGT2B7*, and *UGT2B17*) showed a statistically significant (nominal *p* < 0.05) increase in expression in 38 donors between the ages of 0 and 25 years (**Table 1**). After adjusting for multiple testing, changes in *UGT1A3, UGT1A4, UGT1A5, UGT1A6*, and *UGT2B17* mRNA expression remained significant (FDR < 0.05). It is plausible that the mRNA levels of UGTs are higher after 15 years of age (**Figure 1** shows the expression levels of *UGT1A6* and *UGT2B17* as examples). A *post hoc t*-test used to test the difference in gene expression between younger (0–15 year) and older children (16–25 year) demonstrated that older children have on average >2-fold higher expression levels of most *UGTs* that change with age (*UGT1A1, UGT1A3, UGT1A4, UGT1A5, UGT1A6*, and *UGT2B17*) (*post hoc t*-test, *p* < 0.02 for all tests) (**Table 1**).

**Table 1:** Correlation between age and hepatic UDP-glucuronosyltransferase (UGT) transcription among children.

Gene	β	*p*-value	FDR	^∗^Fold change	^∗^*p*-value
***UGT1A1***	0.354	**0.04**	0.063	2.19	**0.014**
***UGT1A3***	0.514	**0.002**	**0.006**	2.88	**0.015**
***UGT1A4***	0.557	**0.001**	**0.006**	3.17	**0.003**
***UGT1A5***	0.445	**0.008**	**0.018**	2.7	**0.003**
***UGT1A6***	0.513	**0.002**	**0.006**	2.91	**0.014**
***UGT1A9***	0.335	0.053	0.073		
***UGT2B4***	0.224	0.201	0.221		
***UGT2B7***	0.355	**0.039**	0.063		
***UGT2B10***	0.25	0.16	0.196		
***UGT2B15***	0.125	0.471	0.471		
***UGT2B17***	0.521	**0.002**	**0.006**	5.53	**<0.001**

*Both nominal and false discovery rates (FDR)-corrected p-values are shown. Statistically significant values are shown in bold.*

*^∗^Fold changes and p-values were calculated for the post hoc t-test for comparison of mRNA expression levels between young (0–15 years) and old (16–25 years) children groups*.

### Intercorrelation between Age, UGT Expression, and Microsomal Activity

**FIGURE 2:**
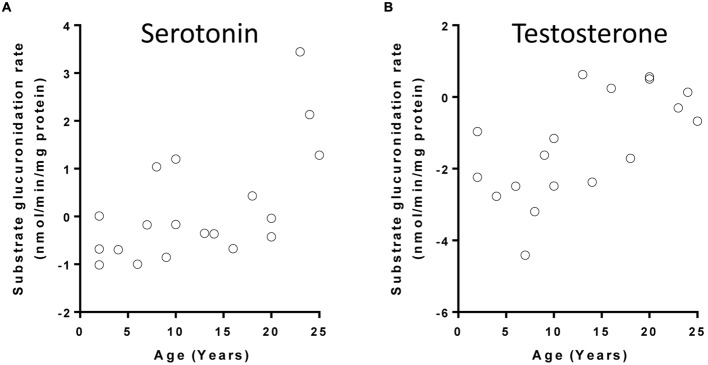
**Correlation between age and UGT substrate glucuronidation rates in children liver microsomes: (A)** serotonin; **(B)** testosterone. Glucuronidation rates (+log2 transformed) of UGT substrates had been previously determined (Kang et al., 2010; Liu et al., 2014).

Microsomal glucuronidation activities of 14 UGT substrates were previously quantified in a subset of the aforementioned liver tissues. The glucuronidation activities of three substrates (serotonin, testosterone, and vorinostat) were nominally associated with age (*p* < 0.05) after controlling for gender (**Table 2**). After FDR adjustment, the change in serotonin glucuronidation remained significant (FDR < 0.05) (**Figure 2** shows serotonin and testosterone glucuronidation as examples). Serotonin is metabolized by UGT1A6, and UGT2B17 is the major UGT involved in the metabolism of both testosterone and vorinostat (Liu et al., 2014). Similar to the gene expression results for *UGT1A6* and *UGT2B17*, children older than 15 years old demonstrated more than twofold higher serotonin and testosterone glucuronidation activity compared to the younger children (*post hoc t*-test, *p* < 0.05, **Table 2**).

**Table 2:** Correlation between age and microsomal glucuronidation activity of UGT substrates.

Drug	β	*p*-value	FDR	^∗^Fold change	^∗^*p*-value	^a^UGT1A6		^a^UGT2B17	
						*r*	*p*	*r*	*p*
**Bilirubin**	0.193	0.467	0.569						
**Serotonin**	0.742	**0.002**	**0.028**	3.78	**0.039**	0.81	**5.40*E*** *-* **05**	0.44	**0.06**
**SN-38**	-0.19	0.488	0.569						
**Flavopiridol**	0.393	0.159	0.334						
**Thyroxine**	0.304	0.267	0.467						
**Epirubicin**	-0.1	0.722	0.778						
**Morphine**	-0.06	0.83	0.830						
***S*-Oxazepam**	-0.2	0.476	0.569						
**Testosterone**	0.609	**0.017**	0.119	2.19	**0.003**	0.21	0.42	0.83	**1.60*E*** *-* **05**
**Mycophenolic acid**	0.416	0.162	0.334						
**Vorinostat**	0.522	**0.047**	0.219						
**Acetaminophen**	0.389	0.153	0.334						
**Anastrozole**	0.281	0.304	0.473						
**Imipramine**	0.377	0.167	0.334						

*The correlation coefficient (r) and p-value of Pearson’s correlation are shown. Statistically significant values are shown in bold.*

*^∗^Fold changes and p-values were calculated for the post hoc t test for comparison of glucuronidation activity between young (0–15 years) and old (16–25 years) children groups.*

*^a^Correlation between *UGT* mRNA expression and microsomal substrate glucuronidation rate among livers*.

To investigate whether the increase in gene expression might cause increased enzyme activity, we further tested the correlations between *UGT1A6* or *UGT2B17* expression and serotonin or testosterone activities, respectively. Strong correlations between *UGT* mRNA expression and their corresponding activities were observed (Pearson’s *r* > 0.8, *p* < 0.0001 for both pairs) (**Table 2**). Both correlations remained significant after controlling for age and sex in a multivariate analysis (*p* < 0.001, data not shown).

### Interrelationship between mRNA Expression of UGTs, TFs, and Age

In order to understand the potential regulatory mechanism underlying the age-related *UGT* transcription, we specifically examined the association between *ESR1* or *PXR* expression and all hepatic *UGT* expression among the children livers. Nominally, ESR1 and PXR were found to be the only TFs whose expression was significantly correlated with age (*p* ≤ 0.05 for both, data not shown). However, this association was not significant after FDR adjustment (FDR > 0.05, data not shown). Nevertheless, there were significant correlations between *ESR1* or *PXR* expression in the pediatric livers and that of nearly every *UGT* gene, even after FDR adjustment (**Table 3**).

**Table 3:** Correlation (Pearson’s correlation) between gene expression of *UGT* and both pregnane X receptor (*PXR)* and estrogen receptor (*ESR1)* among children livers.

UGT genes	*PXR*			*ESR1*		
	*r*	*p*	FDR	*r*	*p*	FDR
*UGT1A1*	0.503	**0.001**	**0.0018**	0.375	**0.02**	**0.022**
*UGT1A3*	0.299	0.073	0.081	0.287	0.085	0.085
*UGT1A4*	0.521	**0.001**	**0.0018**	0.597	**7.6*E*** *-* **05**	**2.8*E*** *-* **04**
*UGT1A5*	0.455	**0.004**	**0.0055**	0.553	**3.2*E*** *-* **04**	**8.8*E*** *-* **04**
*UGT1A6*	0.619	**4.4*E*** *-* **05**	**4.8*E*** *-* **04**	0.448	**0.005**	**0.0092**
*UGT1A9*	0.455	**0.004**	**0.0055**	0.431	**0.007**	**0.0096**
*UGT2B4*	0.548	**3.7*E*** *-* **04**	**0.0014**	0.43	**0.007**	**0.0096**
*UGT2B7*	0.557	**2.8*E*** *-* **04**	**0.0014**	0.672	**4.0*E*** *-* **06**	**2.20*E*** *-* **05**
*UGT2B10*	0.503	**0.001**	**0.0018**	0.724	**4.2*E*** *-* **07**	**4.62*E*** *-* **06**
*UGT2B15*	0.171	0.305	0.305	0.468	**0.003**	**0.0066**
*UGT2B17*	0.318	0.055	0.067	0.38	**0.02**	**0.022**

*Correlation coefficient (r), p-value and FDR-corrected p-value are shown. Statistically significant values are shown in bold.*

## Discussion

The ontogenic expression and regulation of UGT genes remain incompletely understood. We performed a comprehensive survey of the age-dependent mRNA expression and activity of UGTs in livers of children from 0 to 25 years of age. Our study suggested that regulation of a few UGTs (in particular *UGT1A6* and *UGT2B17*) is significantly impacted by age, and these changes may be partly driven by hormonal signaling during development. While many other factors also impact the expression and activity of UGT enzymes (such as medications, supplements, alcohol consumption, and smoking), our findings may help explain some of the inter-patient variability in drug metabolism in pediatric populations.

Ontogeny of UGTs in humans has been explored previously, particularly between pre- and post-natal livers and between children and adult human livers (Burchell et al., 1989; Strassburg et al., 2002; Zaya et al., 2006; Miyagi and Collier, 2007, 2011; Miyagi et al., 2012; Divakaran et al., 2014). For the first time to our knowledge, our study explored the age-dependent mRNA expression and microsomal activity of all major hepatic UGTs in a single sample set with an age range encompassing both childhood and adolescence. We found that a few *UGTs*, especially *UGT1A6* and *UGT2B17*, have more significant age-dependent transcription. This is consistent with the correlation between age and level of glucuronidation activity of UGT1A6 and UGT2B17. These age dependent changes in UGT expression and activity are particularly observed between the childhood group (0–15 years) and adolescent or young adult group (16–25 years), with a more than twofold change on average in both expression and enzyme activity observed between the two age groups. This indicates that pharmacokinetics of therapeutic drugs that are substrates of UGT1A6 and UGT2B17 may change with age, and younger children may have slower intrinsic clearance of these drugs.

Previous studies have demonstrated that protein expression or activities of a few hepatic UGTs are significantly related to age, e.g., UGT1A1, UGT1A4, UGT1A6, UGT2B7, UGT1A9, and UGT2B15 (Zaya et al., 2006; Miyagi and Collier, 2007, 2011; Miyagi et al., 2012; Divakaran et al., 2014). However, major changes of these enzyme expression or activity were observed among the early stage of the development. Our samples are mainly children older than 1 year of age, which does not allow for an assessment for the changes at the early developmental stage. Zaya et al. (2006) observed a significant increase in both protein expression and enzyme activity of UGT2B7 along with age in children across the entire developmental process, we also observed a weak correlation between age and *UGT2B7* mRNA expression in our samples (nominal *p* = 0.039, **Table 1**), though this correlation disappeared after controlling for multiple testing (FDR = 0.063, **Table 1**). We also did not observe a significant correlation between the enzyme activity on epirubicin glucuronidation (*p* = 0.72, **Table 2**). This may again reflect the limitation of our small sample size. However, to our knowledge, it is for the first time that we observed *UGT2B17* expression and activity are positively correlated with age in children.

It should be noted that UGT expression and enzymatic activity are also significantly influenced by genetic variation. This is particularly important for *UGT2B17* where a polymorphism known as copy number variation (CNV) affects the enzyme’s transcription and activity. Our previous study also identified novel regulatory single nucleotide polymorphisms (SNPs) for *UGT2B17* in addition to the CNV polymorphism (Liu et al., 2014). Thus, the regulation of *UGT2B17* transcription among children can be affected by both age and genetic variation. Other than these major factors that are involved in intrinsic drug clearance, the degree of physiological maturation especially organ size and blood flow are also important factors affecting drug pharmacokinetics in children. Therefore, all these factors should be considered for pharmacokinetic and pharmacodynamic analyses of relevant drugs in children.

We observed that PXR and ESR1 are two TFs that modulate *UGT* gene expression in people between ages of 0–25 years, though the association is weak after FDR correction for multiple testing. This weak association may be due to the limited sample size in our study. Our previous study in 125 livers encompassing the entire age range of a human lifespan (0–81 years) demonstrated that PXR and ESR1 are highly important transcriptional regulators of hepatic *UGTs* (Liu et al., 2014). Both TFs play a key role in sensing steroids and xenobiotics (i.e., estrogen and progesterone as well as their metabolites) during transcription of numerous genes, especially those involved in drug/xenobiotic metabolism of hormonal levels. Our data indicate that hormonal regulation may be a critical factor in mediating the age-dependent expression of hepatic UGTs during human development, given the known age-dependent increase in steroid levels. It is known that progesterone is the precursor of both estradiol and testosterone, and both estradiol and progesterone levels significantly increase during puberty in both boys and girls (Fadalti et al., 1999; Ankarberg-Lindgren and Norjavaara, 2008). This is consistent with our observations about changes in UGT expression and enzymatic activity before and after approximately 15 years old. Therefore, the sex hormone signaling may be at least partly involved in the regulatory mechanism underlying age-related *UGT* transcription.

Our study was limited by a small sample size, which decreased our power to detect the ontogenic changes of UGTs, especially in early childhood, e.g., the neonates. The small sample size also limited our capacity to investigate the interaction between genetic and other factors, as well as the impact that this interaction might have on UGT expression and activity. As both genetic alleles and age are known to regulate *UGT* expression in the liver, elucidating the role of this interaction will further establish the key matrix of factors that can be used to determine the pharmacokinetics and pharmacodynamics of UGT substrates in pediatric populations. Our data thus warrant further investigation of these factors within a larger and more diverse population.

## Author Contributions

EN and HM drafted the manuscript and participated in data analysis. JR collected drug metabolism data and participated in manuscript drafting; SM collected gene expression data. MZ established the statistical models. WL conceived the study, analyzed the data, and finalized the writing of the manuscript.

## Conflict of Interest Statement

The authors declare that the research was conducted in the absence of any commercial or financial relationships that could be construed as a potential conflict of interest.
